# Which outcome expectancies are important in determining young adults’ intentions to use condoms with casual sexual partners?: a cross-sectional study

**DOI:** 10.1186/1471-2458-13-133

**Published:** 2013-02-13

**Authors:** Katie V Newby, Katherine E Brown, David P French, Louise M Wallace

**Affiliations:** 1 Applied Research Centre in Heath and Lifestyle Interventions (ARC-HLI), Coventry University, Priory Street, Coventry, CV1 5FB, UK; 2 School of Psychological Sciences, University of Manchester, Manchester, M13 9PL, UK

**Keywords:** Outcome expectancies, Condom use, Theory of planned behaviour, Attitude, Expectancy-value muddle, Dimensional salience

## Abstract

**Background:**

The prevalence of unwanted pregnancy and sexually transmitted infection amongst young adults represents an important public health problem in the UK. Individuals’ attitude towards the use of condoms has been identified as an important determinant of behavioural intentions and action. The Theory of Planned Behaviour has been widely used to explain and predict health behaviour. This posits that the degree to which an individual positively or negatively values a behaviour (termed ‘direct attitude’) is based upon consideration of the likelihood of a number of outcomes occurring (outcome expectancy) weighted by the perceived desirability of those outcomes (outcome evaluation). Outcome expectancy and outcome evaluation when multiplied form ‘indirect attitude’. The study aimed to assess whether positive outcome expectancies of unprotected sex were more important for young adults with lower safe sex intentions, than those with safer sex intentions, and to isolate optimal outcomes for targeting through health promotion campaigns.

**Methods:**

A cross-sectional survey design was used. Data was collected from 1051 school and university students aged 16–24 years. Measures of intention, direct attitude and indirect attitude were taken. Participants were asked to select outcome expectancies which were most important in determining whether they would use condoms with casual sexual partners.

**Results:**

People with lower safe sex intentions were more likely than those with safer sex intentions to select all positive outcome expectancies for unprotected sex as salient, and less likely to select all negative outcome expectancies as salient. Outcome expectancies for which the greatest proportion of participants in the less safe sex group held an unfavourable position were: showing that I am a caring person, making sexual experiences less enjoyable, and protecting against pregnancy.

**Conclusions:**

The findings point to ways in which the attitudes of those with less safe sex intentions could be altered in order to motivate positive behavioural change. They suggest that it would be advantageous to highlight the potential for condom use to demonstrate a caring attitude, to challenge the potential for protected sex to reduce sexual pleasure, and to target young adults’ risk appraisals for pregnancy as a consequence of unprotected sex with casual sexual partners.

## Background

Young adults often engage in unprotected sex despite knowledge of the health related dangers [1-3]. Furthermore, young adults’ sexual risk behaviour appears to be more driven by their perceptions of the positive rather than the negative consequences of unprotected sex [4-9]. Measures assessing the negative consequences of unprotected sex may therefore fail to offer substantial predictive utility with regard to young adults’ sexual risk-taking. The greater focus on the positive consequences by young adults may be explained by the increased likelihood that they have experienced the benefits of unprotected sex but not yet any costs [7].

One of the most commonly used theories in health psychology, which has good predictive utility in the context of condom use [10], is the theory of planned behaviour (TpB) [11,12]. This theory proposes that any behaviour is determined by an individual’s intention to perform that behaviour and perceived behaviour control (PBC), which refers to their perceived ability to perform that behaviour. Intentions are in turn determined also by PBC, and in addition Subjective Norms and Attitudes. Subjective Norms refers to the perceived social pressure to perform or not perform the behaviour, and Attitudes reflects the degree to which the behaviour is positively or negatively valued. An individual’s overall attitude is assessed directly using semantic differential scales, i.e. rating scales with contrasting adjectives at endpoints such as extremely good and extremely bad. According to the TpB, attitude towards a behaviour is based upon consideration of the likelihood of a number of outcomes occurring (termed either behavioural belief or outcome expectancy), weighted by the perceived desirability of those outcomes (termed either value or outcome evaluation). A mixture of positive and negative outcomes is likely to be taken into account in the formation of attitudes. For example, attitude towards using condoms during sexual intercourse may include consideration of the likelihood and value of protecting against sexually transmitted infections (STIs) and pregnancy, but also of reducing sexual pleasure or causing an interruption to sex. The measure of attitude derived using semantic differential scales is termed the “direct” measure, and the measure derived by multiplying outcome expectancies and outcome evaluations is termed the “indirect” measure.

Meta-analyses by Conner and Sparks [13], and McEachan, Conne, Taylor, and Lawton [14] examining the predictive power of the TpB, have found attitude to be a consistently strong determinant of health behaviour with 13.0% and 12.6% of variance explained respectively. This is also true when the TpB has been applied specifically to condom use [10,15]. This suggests that it is important to identify which outcome expectancies are of greatest salience in predicting attitude towards unprotected sex. Most studies examining this theory however do not assess which outcome expectancies are most prevalent and most strongly associated with an individual’s intention and behaviour. This knowledge is crucial in determining which outcome expectancies should be the focus of behavioural change programmes [16,17].

According to the author of the TpB, a measure of indirect attitude is formed by multiplying respondent’s outcome expectancy ratings by their outcome evaluation ratings [18]. This is represented by the following equation:

Attitude=∑bxe

Where *b* = outcome expectancy and *e* = outcome evaluation.

Despite this, assessing indirect attitude presents a unique problem which has been termed the ‘expectancy-value muddle’ [19]. When the outcome expectancy and outcome evaluation scores are multiplied to create ‘multiplicative composites’, the total score calculated is affected by whether unipolar (e.g. 1 to 7) or bipolar (−3 to +3) scoring for each scale, or a combination of the two, are used. Take for example an individual who believed that an outcome was both unlikely to eventuate and undesirable. If a unipolar scale was used for outcome expectancy and a bipolar scale was used for outcome evaluation, then that individual would receive a mid-ranking score (1 x −3 = −3). If however bipolar scales were used for outcome expectancy and outcome evaluation, then that individual would receive the highest score possible (−3 x −3 = 9). The ranking of expectancy-value scores is therefore dependent on the method of scaling used. Results have been shown to vary widely according to which scales are adopted [20-22] suggesting that findings based on the ‘*b* x *e*’ computation are uninterpretable.

A number of different approaches have been suggested to resolve the expectancy-value muddle. One of the most promising approaches, because it avoids use of a multiplicative term, lies in eliciting from participants those outcome expectancies which they consider to be personally salient [19]. The rationale for this approach lies in Cronen and Conville’s [23] assumption that individuals will only produce outcome expectancies that they consider likely to eventuate. As such, the expectancy-value construct can be assessed by asking participants to rate the desirability of outcome expectancies that they have nominated as salient [19] thus avoiding use of a rating scale for the likelihood dimension. The generation and analysis of individually salient items however has some practical limitations. These include maintaining response consistency across items [24], the time-consuming nature of analysing individually-elicited salient outcome expectancies [19], and participants finding the process confusing and difficult to perform [24-26].

Van der Pligt and colleagues [e.g. 27] have proposed a direct method of assessing importance, termed ‘dimensional salience’, which can be used as an alternative to asking participants to produce salient outcome expectancies. They suggested asking participants to provide expectancy and value ratings as normal, then to simply nominate three or five outcome expectancies that they consider to be most important. They showed through a series of studies, that assessing outcome expectancy salience in this way could result in a measure of indirect attitude that outperforms the full set in predicting direct attitude [24,26-31]. This task has been identified as the optimal method because it is time efficient, cognitively undemanding, and because it creates a composite indirect attitude score that performs at least as well as other alternatives in predicting direct attitude and behaviour [32]. Given the constraints of human short-term memory and on-line information processing, a measure of attitude based on a small set of outcome expectancies is also more likely to reflect the actual decision making process [33].

Van Herreveld, van der Pligt and de Vries [34] have examined the structure of young adults’ attitudes towards condom use using the dimensional salience approach. Using a sample of 78 students, they measured direct attitude, indirect attitude, and intention to use condoms during casual sex. They also asked participants to select from a list of 15 positive and negative outcome expectancies for condom use, the five outcome expectancies which they considered would be most important in influencing their behaviour. They found that participants with safe and less safe intentions to use condoms were equally likely to select all positive health-related outcome expectancies for condom use, such as preventing pregnancy and STIs, as salient. Furthermore, they found that those with less safe intentions were more likely to select two negative outcome expectancies for condom use as salient, namely ‘making sex less intimate’ and ‘making sex less comfortable’. They also found that an indirect attitude score based on salient outcome expectancies was as closely related to direct attitude and intention as that based on all outcome expectancy items. Their examination of the utility of the indirect attitude score based on salient outcome expectancies was however tested using the problematic expectancy-value multiplicative composites. The small sample size also limits the strength of conclusion which can be drawn due to a lack of statistical power.

Newton, Ewing, Burney and Hay [35] have assessed the value of using dimensional salience to resolve the expectancy-value muddle in a study examining participant’s outcome expectancies for organ donation. Using a sample of 309 Australian residents, they measured direct attitude and indirect attitude towards posthumous organ donation, and intention to consent to posthumous organ donation. They also asked participants to select from a list of 18 outcome expectancies, those which they considered would be most important in influencing their behaviour. They found the dimensional salience approach to satisfy the Cronen and Conville [23] assumption that outcome expectancies selected as salient would be more likely to eventuate than outcome expectancies that were not selected. This suggested that salient outcome expectancies could be used to represent the expectancy term in expectancy-value models. They also found that an indirect measure of attitude based on salient outcome expectancies only, was more strongly associated with directly assessed attitude and intention, compared to that based on non-salient outcome expectancies only or all outcome expectancies. Finally, they isolated outcome expectancies to be targeted through health promotion campaigns through a process of first identifying outcome expectancies that a greater proportion of undecided than willing consenters held as salient, and second, identifying which of these were held unfavourably by a substantial proportion of undecided consenters. This enabled them to identify important ways in which the outcome expectancies of those willing to consent to organ donation differed from those who were undecided. This approach is of value to public health as it takes the research further in enabling direct recommendations for practice to be made about how to change unfavourable attitudes amongst the population least likely to perform the desired behaviour.

The present study uses the dimensional salience approach, similarly to Newton et al. [35], but in the context of condom use with casual sexual partners. In doing so it overcomes the expectancy-value problem, and enables outcome expectancies to be targeted through health promotion to be drawn out. It examines whether a measure of indirect attitude based on a subset of selected salient outcome expectancies has better utility compared to the full set.

Like van Herreveld, et al. [34] the present study also examines whether negative outcome expectancies associated with protected sex are more likely to be selected by those with less safe than more safe sex intentions to use condoms, and also whether positive outcome expectancies are equally likely to be selected as important. The present study however takes this work forward by employing a large sample to provide much greater confidence in the relationships estimated, and by further examining the effect of gender on outcome expectancy selection. This is important because evidence indicates that gender predicts condom use [36-38] and attitudes towards condom use [39], and that males and females are differentially motivated by the outcomes of unprotected sex [40-42].

The study population was young adults aged 16–24 years from the UK as this age group carries the greatest burden of STI infection [43] and the highest proportion of teenage conceptions is observed amongst those aged 16–18 years old [44]. The focus on condom use with *casual* sexual partners was chosen as it provides an explicit definition of the behaviour under examination. This is important as people’s intentions to use condoms may vary depending on the type of partner, for example casual sexual partners compared to more established long-term partners.

## Method

### Participants

A total of 1414 individuals completed the questionnaire. Incomplete questionnaires were returned by 363 participants who were excluded from the analysis. All analyses reported are based on the remaining 1051 participants. Analysis of responders versus non-responders using chi-squared tests indicated that there was an under-representation of males and some ethnic minority groups in the included group compared to the excluded group. Supplementary analyses exploring the implications of this are reported in Additional file 1: Appendix A.

Of the 1051 participants, 105 were secondary school pupils and 946 were university students. The disproportionately high number of university students in comparison to school pupils was due to an unexpectedly high response rate from university students. A decision was made to include the school pupils to enable representation from the lower end of the at-risk age group, and because a repeat of all main analyses showed their exclusion did not affect the main findings.

Participants ranged from 16–24 years of age; 396 (37.7%) were male, 655 (62.3%) were female. Ethnic breakdown was as follows: White n = 667 (63.5%), South Asian n = 140 (13.3%), Mixed n = 157 (14.9%), Black n = 50 (4.8%), Other n = 36 (3.4%). Ethnic minorities were more greatly represented in this sample (36.5%) than in the English population (9.1%) [45].

### Design

A cross-sectional survey design was used.

### Procedure

The study received Coventry University ethics approval. The questionnaire stated clearly that in this context, ‘sex’ referred to heterosexual sex, that is, ‘penis in vagina’. The term ‘sexually experienced’ is used here to refer to anyone who has ever had sex. A casual sexual partner was defined as ‘someone who you may have sex with, either as a one-night-stand or on a more regular basis, but are not serious about’. Data was collected from both secondary school pupils and university students in order to achieve a sample across the desired age range.

At the school, questionnaires were distributed to pupils in years 12 and 13 within a tutor group period. Pupils were not offered an incentive to participate at the request of the school. Participants were read a participant information sheet to inform them that the questionnaires were anonymous and that they could leave out any questions that they did not wish to answer or choose not to complete the questionnaire at all. Written informed consent was obtained. Completion of the questionnaires took approximately 40 minutes. A written quiz was provided for those who were either ineligible to participate or did not wish to take part, and to occupy those who finished early.

University students were invited to participate using an advert placed on their online learning platform. Those who met the eligibility criteria and were interested in participating were asked to follow a link to a web survey. Students were offered entry into a prize draw to win £100 worth of high street vouchers as an incentive to participate. The web survey was preceded by an information sheet and consent form.

### Measures

Intention to use condoms with casual sexual partners was assessed by three items (intend to, plan to, and try to) using a 7-point response scale ranging from extremely likely to extremely unlikely. These three items were combined to provide a direct assessment of intention (α = .95).

Direct attitude towards condom use with casual sexual partners was assessed by five items, again using 7-point rating scales. The five items were anchored by harmful/beneficial, pleasant/unpleasant, good/bad, worthless/valuable and enjoyable/unenjoyable. These five items were combined to provide a direct assessment of attitude (α = .76).

Indirect attitude was measured by asking participants to provide outcome expectancy (b) and outcome evaluation (e) ratings for 10 possible outcomes of protected sex with casual partners (20 items in total). These items were generated following a review of the literature. This review included identifying papers which reported on outcomes of protected sex derived from elicitation studies. Nine items which captured the most commonly elicited outcomes were selected. The outcome, ‘protecting against chlamydia’ was separated from ‘Other STIs’ to create a tenth item, as evidence suggests that young people may perceive this infection as less important than other health outcomes [46]. The full list of outcomes is presented in table two. Outcome expectancy was assessed using a 7-point scale ranging from 1 (extremely likely) to 7 (extremely unlikely). Outcome evaluation was assessed using a 7-point scale ranging from 1 (extremely desirable) to 7 (extremely undesirable). A score was created for each respondent by summing the b x e products.

Finally, participants were asked to indicate from the pool of ten outcome expectancies, the five most important influences on whether or not they would use condoms with casual sexual partners. The items contained five positive outcome expectancies for protected sex and five negative outcome expectancies. All positive outcome expectancies except one (‘showing that I am a caring person’) were outcome expectancies relating to health such as ‘protecting against pregnancy’. All negative outcome expectancies were hedonistic in nature such as ‘reducing my sexual pleasure’.

### Analysis

The Cronen and Conville [23] assumption was assessed by comparing participants’ mean outcome expectancy scores for outcomes selected as salient and outcomes that were not selected as salient using a paired *t*-test.

Correlation analysis was performed to examine the relationship of intention and direct attitude with three different measures of indirect attitude calculated using participants’ ratings of the desirability of outcome expectancies. The three measures of indirect attitude were based on: (a) all outcome evaluation scores (∑*e*_total_), (b) selected (and therefore salient) outcome evaluation scores (∑*e*_salient_), and (c) unselected (and therefore non-salient) outcome evaluation scores (∑*e*_non-salient_). To protect against inflated type 1 error, a more conservative critical value for detecting significant differences (p = 0.01) was used. The significance of observed differences in the size of correlations between the indirect attitude scales (based on (a) all outcome evaluation scores and (b) salient outcome evaluation scores only) and directly-assessed measures of attitude and intention, was tested using a *t*-test procedure [47].

In order to test for differences between participants with more safe versus less safe sex intentions, the sample was split into two groups so that those with a score of 15 or more on the assessment of condom use intention were termed ‘more safe’ and those with a score of 14 or below were termed ‘less safe’. The minimum possible score was 3 and the maximum was 21. Eighty percent of participants fell into the more safe sex group. Chi-squared analysis was used to compare the proportions of participants who had selected each outcome expectancy for protected sex as one of their five personally salient items. Finally, multi-way frequency tables were produced to identify instances where differing proportions of males and females had selected an outcome expectancy as salient within either the more safe or less safe sex group. Where this indicated that outcome expectancy selection differed across genders, logistic regression analysis was performed. Outcome expectancy salience was the dependent variable. The independent variables gender and intention to have safe sex were included along with the interaction term (gender by intention to have safe sex).

## Results

### Assessing the Cronen and Conville assumption

As participants evaluated their beliefs on a 7-point scale ranging from 1 (extremely unlikely) to 7 (extremely likely), outcome expectancies perceived as likely to eventuate had scores above 4 (the midpoint) and outcome expectancies perceived as unlikely to eventuate had scores at or below 4. Results indicated that participants perceived their salient outcome expectancies (*M* = 5.62, *SD* = 1.00) to be more likely to eventuate than their non-salient outcome expectancies (*M* = 3.85, *SD* = 1.38), *t*(984) = 31.99 *p* < 0.001.

### Predicting attitude and intention

Correlations showing the relationships between intention, directly assessed attitude, and the belief-based measures of indirect attitude are presented in Table 1. Directly assessed attitude correlated more strongly with ∑*e*_salient_ (*r* = .26) than with ∑*e*_total_ (*r* = .15), *t* = 3.25, *df* = 3, p = 0.02. ∑*e*_non-salient_ had a negative correlation with attitude (*r* = −.20). Similarly, intention correlated more strongly with ∑*e*_salient_ (*r* = .21) than ∑*e*_total_ (*r* = .11), *t* = 2.88, *df* = 3, *p* = 0.03. Once more, ∑*e*_non-salient_ had a negative correlation with intention (*r* = −.13). 

**Table 1:** Correlations between the belief-based measures, attitude and intention to use condoms with casual sexual partners

	** A**	** B**	** C**	** D**	** E**
Intention (A)	1.00	.58***	.11***	.21***	-.13***
Direct attitude score (B)		1.00	.15***	.26***	-.20***
Mean score based on all outcome expectancies ∑*e*_total_ (C)			1.00	.35***	.44***
Mean score based on salient outcome expectancies ∑*e*_salient_ (D)				1.00	.50***
Mean score based on non-salient outcome expectancies ∑*e*_non-salient_ (E)					1.00

Note: ***p < 0.001.

### Group differences in salient beliefs

Mean intention to use condoms with casual sexual partners was 20.39 (*SD* = 1.37; *n* = 840) for the more safe group and 11.29 (*SD* = 4.82; n = 211) for the less safe group (max possible score for intention = 21). Chi-square analysis revealed that all outcome expectancies for protected sex were differentially salient for those with more safe and less safe condom use intentions (Table 2). Those in the more safe sex group were more likely than those in the less safe sex group to nominate all positive outcome expectancies for protected sex as salient. Conversely, those in the less safe sex group were more likely than those in the more safe sex group to nominate all negative outcome expectancies as salient

**Table 2:** The proportion of outcome expectancies held as salient by safe and less safe condom users

**Belief item**	**% salient**		***X***^**2**^
	**Safe**	**Less safe**	
***Positive consequences of protected sex***			
Showing that I am a caring person	50.6	29.9	29.16***
Protecting against chlamydia	93.2	72.0	77.02***
Protecting against HIV	96.2	77.3	86.02***
Protecting against other STIs	92.6	72.0	70.12***
Protecting against pregnancy	90.9	64.5	95.56***
***Negative consequences of protected sex***			
Making my sexual experiences less romantic	7.30	21.8	38.98***
Making my sexual experiences less enjoyable	13.3	37.4	65.91***
Causing a annoying interruption	23.2	34.1	10.59***
Reducing my sexual pleasure	13.5	35.1	53.89***
Reducing my partners sexual pleasure	11.7	31.8	51.41***

Note: ***p < 0.001.

Multi-way frequency tables for each of the outcome expectancies were produced to examine whether there were any potential interaction effects between gender and salience for the more safe and less safe sex groups. Examination of the tables revealed that two outcome expectancies, namely ‘causing an annoying interruption to sex’ and ‘protecting against chlamydia’ may be differentially salient for young men and women. Accordingly multiple logistic regression analyses were run for these beliefs (Tables 3 and 4).

**Table 3:** Logistic regression to predict condom use intentions including the gender by ‘interruption to sex’ interaction term

**Outcome expectancy: causing an annoying interruption to sex**	**Gender**	**% (n) salient**	**Odds ratio**	**95%Confidence interval**	**p value**	**Model**
Safe	Male (n = 314)	16.6 (n = 52)	0.26	0.13–0.52	<0.001	Model: *X*^2^ = 28.66, n = 1051, df = 3, p < 0.001 Nagelkerke R^2^ = 0.04
	Female (n = 526)	27.2 (n = 143)	1.00			
Less safe	Male (n = 82)	43.9 (n = 36)	2.02	1.13–3.62	0.02	
	Female (n = 132)	27.9 (n = 36)	1.00			

**Table 4:** Logistic regression to predict condom use intentions including the gender by ‘not getting chlamydia’ interaction term

**Outcome expectancy: Not getting chlamydia**	**Gender**	**% (n) sali0065nt**	**Odds ratio**	**95%Confidence interval**	**p value**	**Model**
Safe	Male (n = 314)	90.4 (n = 284)	1.51	0.66–3.43	0.33	Model: *X*^2^ = 81.01: n = 1051: df = 3, p < 0.001 Nagelkerke R^2^ = 0.15
	Female (n = 526)	94.9 (n = 499)	1.00			
Less safe	Male (n = 82)	58.5 (n = 48)	0.34	0.18–0.63	<0.001	
	Female (n = 129)	80.6 (n = 104)	1.00			

Amongst those in the more safe sex group, males were approximately 75% less likely than females to select ‘causing an annoying interruption’ as salient. Conversely, amongst those in the less safe sex group, males were twice as likely as females to select this outcome expectancy as salient. Also within the less safe sex group, males were 65% less likely than females to select ‘protecting against chlamydia’ as salient.

## Discussion

Cronen and Conville’s [23] assumption that individuals will only elicit outcome expectancies that they consider likely to eventuate was satisfied by data from the present study. This indicates that outcome expectancy salience can be used instead of the expectancy term in expectancy-value models and further supports use of the dimensional salience approach to resolve the expectancy-value muddle. The indirect attitude score based on salient outcome expectancies was a better predictor of direct attitude and intention than that based on either non-salient outcome expectancies or all outcome expectancies. Participants who reported high levels of intentions to use condoms with casual sexual partners (more safe sex group) were more likely than those in the less safe group to endorse all positive (largely health-related) outcome expectancies for protected sex as salient. Conversely, those in the less safe sex group were more likely than those in the more safe sex group to endorse all of the negative (hedonic) outcome expectancies for protected sex as salient. Males in the more safe sex group were less likely than females to select ‘causing an annoying interruption’ as one of their salient outcome expectancies. Within the less safe sex group, males were less likely than females to select ‘protecting against chlamydia’ as one of their salient outcome expectancies.

This study builds on previous work by van Herreveld et al. [34] in examining the underlying belief structure of young adults’ attitudes towards condom use, and is the first known study to do this within the context of casual sexual relationships. The much larger sample size used in this study increases confidence in the findings and allows an adequately powered comparison between males and females. In adopting the dimensional salience approach, the statistical issue known as the expectancy-value muddle, has been avoided.

Findings should be interpreted in the context of the study’s limitations. As the sample consisted of secondary school and university students, the extent to which the findings can be generalised to the wider population is not known. As this was a cross-sectional study, a measure of future behaviour was not taken. There is potential for the findings to differ in important ways if behaviour rather than intention was considered in the analyses. Although it is accepted that those with strong intentions to use condoms with casual sexual partners may not translate this into action, evidence from a meta-analysis of experimental tests of the intention-behaviour relationship [48] indicates that a medium-to-large change to intention leads to small-to-medium change in behaviour. Given that those with stronger intentions to use condoms in the first place are more likely to go on to use condoms than those with weaker intentions, a better understanding of how to move some of those young people from the less safe sex group into the more safe sex group still has value because this proffers a better chance that motivation will be translated into action. There was an over-representation of ethnic minority participants in the sample compared to the national average [45] which was not adjusted for in the analysis. Whilst this is commensurate with UK cities such as that from which the sample was drawn, care should be taken when generalising to non-urban populations. It is possible for example, that outcome evaluation ratings or selection of salient outcome expectancies are influenced by cultural or religious factors. A proportion of participants were excluded from the analysis due to incomplete data. Analysis of responders versus non-responders identified that there was an under-representation of males and some ethnic minority groups in the included sample compared to the excluded sample. Supplementary analysis reported in Appendix A however, suggests that if response rates had been the same across gender and ethnic groups, the overall pattern of results would be highly similar to those reported.

The results of this research show that including a measure of outcome expectancy salience has the potential to improve understanding of the underlying structure of young adults’ attitudes towards condom use. Our findings show that a measure of indirect attitude based on salient outcome expectancies is more closely related to direct attitude and intentions than that based on non-salient outcome expectancies and that based on all outcome expectancies. This indicates that the measure based on selected salient outcome expectancies is superior to that based on all outcome expectancies, a finding which supports existing evidence [24,26-31].

One of the primary goals of this study was to identify which outcome expectancies were most important in determining young adults’ intentions to use condoms with casual sexual partners. In both the more safe sex and the less safe sex groups, relatively high proportions of participants selected each of the health-related outcome expectancies as important influences on their behaviour. This is consistent with other findings across health behaviours including condom use [34,49]. Those with safer sex intentions were however still more likely to select health related outcome expectancies as important. This suggests that those with less safe intentions may perceive health consequences such as pregnancy and STIs as less of a threat.

Those with less safe sex intentions perceived the negative outcome expectancies for protected sex as more important than those with safer sex intentions. Again, this pattern of results is consistent with previous research [34,49], that is, that individuals have a tendency to perceive outcome expectancies that support their behaviour as more important. The negative outcome expectancies rated in the present study are all hedonic or affective in nature and conversely reflect the positive consequences of unprotected sex. The negative outcome expectancy for protected sex ‘making my sexual experienced less enjoyable’, can for example also similarly be considered as a positive outcome expectancy for unprotected sex, that is ‘making my sexual experiences more enjoyable’. As such, the findings of the present study support existing evidence which indicates that young adults’ risky sexual behaviour is driven by their positive outcome expectancies for unprotected sex [4-9].

Examining the structure of attitudes, and comparing differences between groups with more safe and less safe sex intentions, improves understanding of condom use decision making. In particular, the salient outcome expectancies that underlie the attitudes of young adults who have lower intentions to adopt safe sex practices could act as targets of interventions aiming to improve condom use. This follows the same approach adopted by Newton et al. [35] which was used to isolate optimal outcomes for posthumous organ donation to be targeted through health promotion campaigns. In the present study, all outcome expectancies were found to be differentially salient for those in the more safe and less safe sex groups suggesting that modifying these may be effective in changing attitude and behavioural intention. Hornik and Woolf [50] have however suggested that an additional factor should be taken into account. They propose that interventions should target beliefs for which a substantial proportion of the population have an unfavourable position, a similar position to Ajzen [51] who proposes targeting constructs which have the lowest mean levels. By applying this additional approach to the findings, it is possible to narrow down the full set of outcome expectancies to a smaller group which are optimal for targeting through health promotion campaigns.

All of the health-related outcome expectancies associated with preventing STIs were held by substantial proportions of participants in both the more safe and less safe sex groups suggesting that if targeted, these would be the least efficacious in modifying attitudes and intentions. Beliefs for which the greatest proportions of participants in the less safe sex group held an unfavourable position were in descending order: showing that I am a caring person (held by 49.4% and 70.1% of safe and less safe participants respectively), making sexual experiences less enjoyable (held by 13.3% and 37.4% of safe and less safe participants respectively), and protecting against pregnancy (held by 9.1% and 35.5% of safe and less safe participants respectively). Accordingly, it may be advantageous to address these outcome expectancies as part of health promotion initiatives. This could be done for example, by highlighting the potential for condom use to demonstrate a caring attitude, by challenging and addressing the potential for protected sex to reduce sexual pleasure e.g. through persuasive messages and promoting condoms that maximise sensitivity and comfort, and through targeting young adults’ risk appraisals for pregnancy.

An alternative criterion for determining which outcome expectancies to target through health promotion is to isolate beliefs where there is the greatest absolute difference in the proportion of participants with favourable and unfavourable intentions [16,17,52,53]. If this approach were adopted for the present study, then the greatest absolute difference between the more safe and less safe sex groups is for the outcome expectancy ‘protecting against pregnancy’ (with a difference of 26.4 percentage points), followed by the outcome expectancies ‘making my sexual experiences less enjoyable (with a difference of 24.1 percentage points), and ‘reducing my sexual pleasure’ (with a difference of 21.6 percentage points). This demonstrates that the choice of criterion on which to base decisions about which outcomes expectancies to take forward for targeting can affect the result, although in this case there is a high degree of overlap. A further approach to isolating outcome expectancies for targeting through health promotion campaigns would be to identify at an individual level which outcome expectancies were held least favourably, and then to deliver individually tailored messages. This could be achieved through for example, a web-delivered intervention.

There were two outcome expectancies where the proportion of young adults selecting the outcome as salient differed by gender within the more safe and less safe sex groups. This is commensurate with other studies which have identified that males and females are differentially motivated by the outcomes of protected sex [40-42]. Males in the less safe group were twice as likely as females to select condoms ‘causing as annoying interruption to sex’ as an important influence on whether they would use condoms. The opposite pattern of findings was observed in the more safe sex group. This may reflect the perception that condom use is a gender-appropriate role, with men more likely to take control over their use and therefore to suffer the greatest disruption to their sexual experience. Males in the less safe sex group were also less likely than females to select condoms ‘protecting against chlamydia’ as salient. This finding may reflect the belief that chlamydia only has long-term consequences for women as has been found elsewhere [46]. These findings suggest that targeted work with young males to favourably influence outcome expectancies relating to interrupting sex and protecting against chlamydia may be beneficial in increasing their intentions to use condoms with casual sexual partners. This may be particularly worthwhile for the outcome expectancy ‘causing an annoying interruption to sex’ where a reasonably high proportion of young adults in the unsafe sex group (34.1%) indicated that this outcome was an important influence on their behaviour. It may for example be helpful to suggest ways of incorporating condom use into foreplay to make it less disruptive.

## Conclusions

In conclusion, this study has provided insight into the structure of young adults’ attitudes towards condom use with casual sexual partners. The findings point to ways in which the attitudes of those with less safe sex intentions could be altered in ways that motivate positive behavioural change. There is a tendency for intervention programmes to be developed on the basis of what is known about those who are already motivated to perform the recommended behaviour and also to focus on positive health-related outcome expectancies. Through a better understanding of which outcome expectancies are most salient to those in the target group, including in this case that negative outcome expectancies for protected sex are salient for those with less safe sex intentions, it may be possible to develop more effective health education interventions.

## Competing interests

The authors declare that they have no competing interests.

## Authors’ contributions

KN and DF conceived of the study and participated in its design. KN collected the data, and carried out the statistical analysis. KN, DF, KB and LW drafted the manuscript. All authors read and approved the final manuscript.

## Authors’ information

KN is a Research Fellow and HPC registered Practitioner Health Psychologist.

KB is a Reader in eHealth and Wellbeing interventions, leads a research group studying adolescent sexual health, and a BPS chartered health psychologist. DF is a professor of Health Psychology and BPS chartered health psychologist. LW is a professor of Psychology and Health and BPS chartered health psychologist.

## Pre-publication history

The pre-publication history for this paper can be accessed here:

http://www.biomedcentral.com/1471-2458/13/133/prepub

## Supplementary Material

Additional file 1 Appendix A. Click here for file
