# Supplementary material for: Which outcome expectancies are important in determining young adults’ intentions to use condoms with casual sexual partners?: a cross-sectional study
Source: BMC Public Health. 2013 Feb 13;13:133. doi: 10.1186/1471-2458-13-133 (PMC3599836; doi:10.1186/1471-2458-13-133)
Supplement: Additional file 1 — Appendix A. [file 1471-2458-13-133-S1.docx]

**Appendix A**

As indicated within the main body of the paper, 363 of 1414 participants were excluded from analyses due to the return of incomplete questionnaires. Analysis of responders versus non-responders using chi-squared tests indicated that there was an under-representation of males and some ethnic minority groups in the included group compared to the excluded group. Accordingly further analyses have been conducted to explore the implications of this.

**Assessing the Cronen and Conville [23] assumption**

To test the effect of ethnicity and gender on the difference between these mean outcome expectancy scores, repeated measures ANCOVAs was performed with ethnicity and gender entered as a covariate. There was no evidence that ethnicity had any effect on the difference between these mean outcome expectancy scores (all *p*>0.37). Gender however interacted with the main effect(*F*=13.45, df=1, 978, *p*<0.001). Further analysis was therefore performed examining males and females independently using paired t-tests. The assumption that outcome expectancy scores would be higher for beliefs selected as salient than beliefs not selected as salient held true for females (*t*(619)=28.59 *p*<0.001) and males (*t*(364)=15.92 *p*<0.001), although the effect was larger for females. This indicates that the overall finding would hold true if the excluded sample, which has an over-representation of males and some ethnic minority groups, were included in the analysis.

**Predicting attitude and intention**

Correlation analyses to examine the relationship between ethnicity, gender, intention and direct attitude were performed. Four dummy variables (Black participants v others, Asian participants v others, White v others, and Mixed ethnicity v others) were created to examine ethnicity. Ethnicity did not correlate with intention (all p>0.75) or attitude (all p>0.24). Gender did not correlate with intention (*p*=0.57) but did correlate with direct attitude (*p*<0.001). To further investigate this, multiple regression analyses were performed. Gender was in entered in the first block, each measure of indirect attitude was entered separately in the second block, and then the interaction between these two variables was entered in the final block. The results of these three models are presented in tables A1 to A3 below. All of these analyses indicate that the indirect measures of attitude have a significant effect on direct attitude over and above the effect of gender. In each case, there was no interaction between the indirect measure of attitude and gender (app p>0.11) and the addition of this block did not add significant additional variance over the previous block. Once more, the overall effects remain unchanged by ethnicity and gender, indicating that the exclusion of a proportion of the sample has not affected the pattern of findings.

Table A1. Multiple regression analysis of the relationships of gender, ∑*e*_total_ and their interaction, with direct attitude

|  |  | *b* | *β* | Model |
| --- | --- | --- | --- | --- |
| Block 1  Block 2  Block 3 | Gender  Gender  ∑*e*_total_  Gender  ∑*e*_total_  Gender_*_∑*e*_total_ | -1.30  -1.18  .14  -5.35  .00  .10 | -.24***  -.11***  .14***  -.51*  .00  .42 | N=1044  R^2^ = .02***  AdjR^2^ =.01  R^2^ = .04***  AdjR^2^ =.03  R^2^ = .04***  AdjR^2^ =.04 |
|  |  |  |  |  |
|  |  |  |  |  |

^Note: *p<0.05; **p< 0.01, ***p<0.001^

∑*e*_total is a measure of indirect attitude based on the sum of all outcome expectancy ratings_

Table A2. Multiple regression analysis of the relationships of gender, ∑*e*_salient_ and their interaction, with direct attitude

|  |  | *b* | *β* | Model |
| --- | --- | --- | --- | --- |
| Block 1  Block 2  Block 3 | Gender  Gender  ∑*e*_salient_  Gender  ∑*e*_salient_ Gender_*_∑*e*_salient_ | -1.30  -.99  .10  -1.03  .10  .00 | -.12***  -.09**  .25***  -.10  .24**  .01 | N=1044  R^2^ = .02***  AdjR^2^ =.01  R^2^ = .08***  AdjR^2^ =.07  R^2^ = .08***  AdjR^2^ =.07 |
|  |  |  |  |  |
|  |  |  |  |  |

^Note: *p<0.05; **p< 0.01, ***p<0.001^

∑*e*_salient is a measure of indirect attitude based on the sum of all salient outcome expectancy ratings_

Table A3. Multiple regression analysis of the relationships of gender, ∑*e*_nonsalient_ and their interaction, with direct attitude

|  |  | *b* | *β* | Model |
| --- | --- | --- | --- | --- |
| Block 1  Block 2  Block 3 | Gender  Gender  ∑*e*_nonsalient_  Gender  ∑*e*_nonsalient_ Gender*∑*e*_nonsalient_ | -1.32  -1.39  -.12  -1.83  -.18  .04 | -.12***  -.13***  -.20***  -.17***  -.29**  .10 | N=1044  R^2^ = .01***  AdjR^2^ =.02  R^2^ = .06***  AdjR^2^ =.05  R^2^ = .06***  AdjR^2^ =.05 |
|  |  |  |  |  |
|  |  |  |  |  |

^Note: *p<0.05; **p< 0.01, ***p<0.001^

∑*e*_nonsalient is a measure of indirect attitude based on the sum of all salient outcome expectancy ratings_

**Group differences in salient beliefs**

Interaction effects between gender and salience for the more safe and less safe sex groups are reported on in the main body of the paper as this was a focus of the study. Multi-way frequency tables for each of the outcome expectancies were produced to examine whether there were also any potential interaction effects between ethnicity and salience for the more safe and less safe sex groups. There was evidence that all outcome expectancies may be differentially salient for young people depending on their ethnicity. Accordingly, logistic regression analyses were run for all items, entering outcome expectancy salience as the dependent variable. These analyses showed that the relationships observed between outcome expectancy salience and condom use (safe vrs less safe), as reported in Table 2 of the main document, were attributable to White participants only (all p<0.01). The selection of outcome expectancies did not differ between those in the safe and less safe sex groups for any of the other ethnic groups. Low numbers of ethnic minority participants in the less safe sex group may account for this. In other words, differences may have been observed if there had been a larger proportion of ethnic minority participants in the sample. We cannot know whether this would be the case or if so, whether the pattern of these differences would be the same as for White participants. Irrespective of this, the strong associations between outcome expectancy salience and condom use (see Table 2 in main document) suggest that even if this pattern were different, that the addition of the excluded participants (which had a greater proportion of ethnic minority participants than in the included sample) would not have changed the direction or significance of the association. The same is true for the effect of gender, where the inclusion more males in the sample would not have changed the overall finding.
